# PRRSV-infected monocyte-derived dendritic cells express high levels of SLA-DR and CD80/86 but do not stimulate PRRSV-naïve regulatory T cells to proliferate

**DOI:** 10.1186/s13567-015-0186-z

**Published:** 2015-05-20

**Authors:** Irene M Rodríguez-Gómez, Tobias Käser, Jaime Gómez-Laguna, Benjamin Lamp, Leonie Sinn, Till Rümenapf, Librado Carrasco, Armin Saalmüller, Wilhelm Gerner

**Affiliations:** Institute of Immunology, Department of Pathobiology, University of Veterinary Medicine, Vienna, Veterinärplatz 1, 1210 Vienna, Austria; Department of Anatomy and Comparative Pathology, Faculty of Veterinary Medicine, University of Córdoba, “International Excellence Agrifood Campus – CeiA3”, 14014 Córdoba, Spain; Vaccine and Infectious Disease Organization-International Vaccine Centre (VIDO-InterVac), University of Saskatchewan, 120 Veterinary Road, S7N 5E3 Saskatoon, Saskatchewan Canada; CICAP – Food Research Centre, 14400 Pozoblanco, Córdoba, Spain; Institute of Virology, Department of Pathobiology, University of Veterinary Medicine, Vienna, Veterinärplatz 1, 1210 Vienna, Austria

## Abstract

In vitro generated monocyte-derived dendritic cells (moDCs) have frequently been used to study the influence of porcine reproductive and respiratory syndrome virus (PRRSV) infection on antigen presenting cells. However, obtained results have often been conflicting in regard to expression of co-stimulatory molecules and interaction with T cells. In this study we performed a detailed phenotypic characterisation of PRRSV-infected moDCs and non-infected moDCs. For CD163 and CD169, which are involved in PRRSV-entry into host cells, our results show that prior to infection porcine moDCs express high levels of CD163 but only very low levels for CD169. Following infection with either PRRSV-1 or PRRSV-2 strains after 24 h, PRRSV-nucleoprotein (N-protein)^+^ and N-protein^−^ moDCs derived from the same microculture were analyzed for expression of swine leukocyte antigen-DR (SLA-DR) and CD80/86. N-protein^+^ moDCs consistently expressed higher levels of SLA-DR and CD80/86 compared to N-protein^−^ moDCs. We also investigated the influence of PRRSV-infected moDCs on proliferation and frequency of Foxp3^+^ regulatory T cells present within CD4^+^ T cells in in vitro co-cultures. Neither CD3-stimulated nor unstimulated CD4^+^ T cells showed differences in regard to proliferation and frequency of Foxp3^+^ T cells following co-cultivation with either PRRSV-1 or PRRSV-2 infected moDCs. Our results suggest that a more detailed characterisation of PRRSV-infected moDCs will lead to more consistent results across different laboratories and PRRSV strains as indicated by the major differences in SLA-DR and CD80/86 expression between PRRSV-infected and non-infected moDCs present in the same microculture.

## Introduction

Porcine reproductive and respiratory syndrome (PRRS) is one of the most devastating diseases for the swine industry world-wide [1,2]. The causative agent, PRRS-virus (PRRSV), belongs to the genus *Arterivirus* and comprises two different genotypes designated as type 1 (PRRSV-1) and type 2 (PRRSV-2) (formerly European and American genotypes, respectively) [3,4]. PRRSV is able to persist in the host for a long period of time [5-7] supported by a delayed onset of specific humoral and cellular immune responses [8,9].

The mechanisms of this delay are still under investigation. One possible reason could be a decreased stimulation of CD4^+^ T cells by antigen presenting cells (APCs) which play a central role in T-cell activation via the expression of MHC-II and CD80/86 [10]. In order to study the role of these molecules in PRRSV infection a frequently used model are in vitro generated cultures of monocyte-derived dendritic cells (moDCs). However, published findings on expression levels of MHC-II and CD80/86 expression in PRRSV-infected moDC cultures have led to conflicting results. Some reports claim no changes [11] or a decreased [12-14] expression of swine leukocyte antigen (SLA)-DR and a decrease [13] or increase [14] of CD80/86 expression on infected moDCs with either PRRSV-1 or PRRSV-2 strains.

Another possible explanation for the ineffective adaptive immune response may be the activation or induction of regulatory T cells (Tregs) by PRRSV. Indeed, it has been shown that several viruses such as the human immunodeficiency virus, hepatitis C virus or feline immunodeficiency virus use the induction of Tregs in order to suppress or evade the immune response by the host (reviewed in [15]). Since the first description of porcine Tregs [16] and the analysis of their suppressive capabilities [17] much work was devoted to the study of this T-cell subset during PRRSV infection. Some reports showed an in vitro induction of Tregs within peripheral blood lymphocytes (PBLs) after co-cultivation with PRRSV-2 infected moDCs [18,19]. However, this was not the case when several PRRSV-1 strains [11] and a high-virulent PRRSV-2 strain (VR2385) were tested [20]. Ex vivo analyzes of Tregs following PRRSV-2 infection of nine week old pigs also indicated an increase of CD4^+^CD8α^+^Foxp3^+^ Tregs [21].

Due to the controversial results of the effect of PRRSV-infection onto APCs and onto their MHC-II and CD80/86 expression, one of the aims of this study was to assess changes on these two molecules using moDCs infected with PRRSV-2 and PRRSV-1 strains. In comparison to previous reports, we focus on differences between PRRSV-infected and non-infected moDCs present in the same microculture. Furthermore, by making use of the same culture system we revisited the effect of PRRSV-infected moDCs on Foxp3 expression and proliferation of CD4^+^ T cells in co-culture experiments.

## Materials and methods

### Animals and peripheral blood mononuclear cells (PBMCs) isolation

Six-month old crossbred (Large White X Landrace X Pietrain) pigs from an abattoir served as blood donors for the isolation of PBMCs. The general health status of all animals was controlled before transportation and after arrival at the slaughter plant and all animals appeared clinically healthy. The pigs were subjected to electric high voltage anesthesia followed by exsanguination. This procedure is in accordance to the Austrian Animal Welfare Slaughter Regulation. Heparinized blood was collected at the moment of slaughter.

PBMCs were isolated by density centrifugation with Pancoll (density 1.077 g/mL; PAN Biotech, Aidenbach, Germany) as described elsewhere [22]. Collected PBMCs were washed and resuspended in complete medium (CM; RPMI 1640 with stable L-Glutamine supplemented with 10% heat-inactivated fetal calf serum (FCS), 100 IU/mL penicillin and 100 μg/mL streptomycin; all from PAN Biotech) in a 75 cm^2^ tissue culture flask (Greiner Bio-One, Kremsmünster, Austria) for subsequent generation of moDCs.

During PBMC isolation process, plasma samples were collected and tested for antibodies against PRRSV (IDEXX PRRS X3 Ab Test, IDEXX Europe B.V., Hoofddorp, The Netherlands) by an external laboratory for veterinary diagnostics (LaboVet, Vienna, Austria). Only PBMCs from PRRSV antibody-negative donors were used in the subsequent experiments.

### Generation of moDCs

Monocytes were separated from PBMCs by plastic adherence for 90 min at 37 °C in 5% CO_2_. Afterwards, PBLs were removed and immediately frozen for long-term storage at −150 °C as described by Leitner et al. [23]. The remaining plastic-adherent cells were washed twice with CM. MoDCs were generated as previously described by Carrasco et al. [24] with minor modifications. Briefly, plastic-adherent monocytes were cultured in CM supplemented with 40 ng/mL of recombinant porcine (rp) GM-CSF (R&D Systems, Minneapolis, MN, USA) and 40 ng/mL of rpIL-4 (R&D Systems) at 37 °C in 5% CO_2_. After three days the medium was replaced by fresh cytokine-supplemented CM. Seven days after the start of in vitro cultivation moDCs were harvested with a cell scraper (Greiner Bio-One).

### Viruses

Two different PRRSV strains belonging to type 1 and 2 of PRRSV were used in this study. A European PRRSV-1 field isolate was propagated on MA-104 cells. This isolate showed 87% homology with the European genotype subtype 1 Lelystad virus based on genome position 12280–13460 (NCBI GenBank accession number KM657092). Five days post-infection, the supernatant was collected, cleared by centrifugation and passed through a 0.45 μm filter membrane. The PRRSV-2 strain NVSL 97–7895 was rescued after electroporation of 5 × 10^6^ HEK293T cells (0.1 kV, 0.95 mF, 0.2 mm gap cuvette) with 1 μg capped SP6 transcripts from an A*cl*I linearized modified full length clone pFL12 [25] generously provided by Fernando Osorio, University of Nebraska. Three days post transfection the supernatant was collected and cleared by centrifugation. Since NVSL 97–7895 displayed reduced growth kinetics on MA-104 cells, the subclone MARC-145 [26] was used for virus production. Therefore, 5 × 10^6^ MARC-145 cells seeded in 10 cm cell culture dishes were infected with the progeny virus. Five days after infection the supernatant was harvested and used to infect fresh MARC-145 cell cultures. When severe cytopathic effect was observed, the supernatant was collected by centrifugation to serve as virus stock. Virus stocks were stored at −80 °C as aliquots. One aliquot of each strain was thawed and viral titers were determined by titration on MA-104 cells for the PRRSV-1 strain and MARC-145 cells for the PRRSV-2 strain. Despite the initial propagation of the PRRSV-1 strain on MA-104 cells this virus showed comparable titers on both MA-104 and MARC-145 cells (1 × 10^4^ focus forming units on MA-104 and 2 × 10^4^ focus forming units on MARC-145 cells). Multiplicity of infection (MOI) values were calculated from titers on MA-104 cells for the PRRSV-1 strain and MARC-145 cells for the PRRSV-2 strain. All experiments were performed with the same batch of viruses.

### Flow cytometry (FCM)

FCM analysis was carried out on a FACSCanto™ II (BD Biosciences, San Jose, CA, USA). Data were processed by using FACSDiva software (Version 6.1.3., BD Biosciences) and FlowJo software (Version 7.6, Treestar, Ashland, OR, USA). Details on the staining procedures are given below. Incubation steps for labeling of extracellular antigens, including secondary reagents, lasted for 15 min at 4 °C in the fridge and 30 min under the same conditions for intracellular antigens. For washing steps, performed after each incubation, a buffer containing PBS and 2% FCS was used for extracellular antigens. Prior to the use of Live/Dead® Fixable Near-IR (Life Technologies, Carlsbad, CA, USA, see below for details) cells were washed in pure PBS. Wherever appropriate, isotype-matched control samples or fluorescence minus one samples were included in all staining procedures outlined below.

#### Phenotyping of moDCs

Monocytes adhered to culture flasks (day 0), or moDC-cultures after 4 and 7 days of cultivation were harvested and labelled using four different combinations of monoclonal antibodies (mAbs) listed in Table 1. In a first sample mAbs specific for CD1, CD14, and CD172a were combined. A second sample comprised mAbs for CD14, SLA-DR and CD172a; a third mAbs for CD169 and CD172a together with CD152/Fc chimeric protein for labeling of CD80/86. In a fourth sample mAbs for CD163, SLA-DR and CD172a were combined. These samples were labelled in a second incubation step with secondary reagents listed in Table 1 [27,28]. Additionally, during this incubation step Live/Dead® Fixable Near-IR (Life Technologies) was added according to the manufacturer’s instructions.

**Table 1:** **Antibody panels used for FCM analyzes**

**Antigen**	**Clone**	**Isotype**	**Fluorochrome**	**Labeling strategy**	**Source of primary Ab**
*Phenotyping of moDCs*					
CD1	76-7-4	IgG2a	Alexa647	secondary antibody^a^	in house^j^
CD14	CAM36A	IgG1	BV421	secondary antibody^b^	VMRD
CD80/86	CD152/Fc chimera	IgG2a	Alexa647	secondary antibody^a^	Sigma-Aldrich
CD163	2A10/11	IgG1	PE	directly conjugated	AbD Serotec
CD169	3B11/11	IgG1	BV421	secondary antibody^b^	AbD Serotec
CD172a	74-22-15A	IgG2b	Alexa488	secondary antibody^c^	in house^j^
SLA-DR	MSA3	IgG2a	Alexa647	secondary antibody^a^	in house^k^
*Assessment of moDC infection rate*					
CD172a	74-22-15A	IgG2b	PE	secondary antibody^d^	in house^j^
PRRSV-N	P10/b1	IgG1	Alexa488	directly conjugated^e^	in house^l^
*CD163, CD169, SLA-DR and CD80/86 expression on infected moDCs*					
CD172a	74-22-15A	IgG2b	Alexa647	secondary antibody^f^	in house^j^
SLA-DR	MSA3	IgG2a	PE	directly conjugated^g^	in house^k^
CD80/86	hCTLA4/Fc chimera	IgG2a	BV421	two step biotin-streptavidin^h^	Sigma-Aldrich
CD163	2A10/11	IgG1	PE	directly conjugated	AbD Serotec
CD169	3B11/11	IgG1	BV421	two step biotin-streptavidin^i^	AbD Serotec
PRRSV-N	P10/b1	IgG1	Alexa488	directly conjugated^e^	in house^l^

^a^Goat anti-Mouse IgG2a-Alexa647, Life Technologies.

^b^Rat anti-Mouse IgG1-BV421, clone RMG1-1, BioLegend.

^c^Goat anti-Mouse IgG2b-Alexa488, Life Technologies.

^d^Goat anti-Mouse IgG2b-PE, Southern Biotech.

^e^Alexa Fluor® 488 Protein Labeling Kit, Life Technologies.

^f^Goat anti-Mouse IgG2b-Alexa647, Life Technologies.

^g^Lightning-Link™ RPE Conjugation Kit, Innova Biosciences.

^h^Goat anti-Mouse IgG2a-biot., Southern Biotech; Streptavidin-BV421, BioLegend.

^i^Goat anti-Mouse IgG1-biot., Southern Biotech; Streptavidin-BV421, BioLegend.

^j^Pescovitz et al. [27].

^k^Hammerberg and Schurig [28].

^l^Weiland et al. [29].

#### Analysis of moDC infection rate

After seven days of cultivation, moDCs were seeded into round-bottomed 96-well plates (Greiner Bio-One) at 2 × 10^5^ cells per well and infected with PRRSV-1 or PRRSV-2 strains at an MOI of 0.1 at 37 °C in 5% CO_2_. After two hours, the inoculum was removed and cells were washed twice with CM. Separate microcultures with mock-infected moDCs were included in each experiment. Cultivation of moDCs was continued until 12, 24 and 48 hours post-inoculation (hpi). At these time points moDCs were harvested and analyzed for PRRSV-N-protein expression (see below for details). In addition, moDCs were infected with different MOI (0.1, 0.02 and 0.004) and harvested after 24 hpi. Harvested moDCs were labelled with anti-CD172a mAbs (see Table 1 for details on staining strategy) and isotype-specific secondary antibodies in combination with Live/Dead® Fixable Near-IR during a second incubation step. Subsequently, moDCs were fixed and permeabilized by the Foxp3/Transcription Factor Staining Buffer Set (eBioscience, San Diego, CA, USA) according to manufacturer’s instructions. Thereafter, cells were incubated with anti-PRRSV-N-protein mAbs (clone P10/b1) [29]. This antibody had been purified and conjugated to Alexa488 as described elsewhere [30].

#### Expression of CD163, CD169, SLA-DR and CD80/86 on infected moDCs

MoDCs were infected with PRRSV-1 or PRRSV-2 strains at an MOI of 0.02 at 37 °C in 5% CO_2_ under the conditions described above. After 24 hpi, moDCs were harvested and labelled with anti-CD172a mAbs and anti-CD80/86 immunoglobulin fusion proteins (see Table 1 for details) followed by incubation with secondary reagents. Free binding sites of secondary antibodies were blocked with whole mouse IgG (1 μg per sample, ChromPure, Jackson ImmunoResearch, West Grove, PA, USA). Thereafter, mouse anti-porcine SLA-DR-PE mAbs and Live/Dead® Fixable Near-IR were added. In parallel samples CD80/86-specific fusion proteins and SLA-DR-specific mAbs were replaced by mAbs against CD163 and CD169. Details on the labeling strategy of CD163 and CD169 are provided in Table 1. Lastly, cells were fixed and permeabilized and labelled for PRRSV-N-protein expression as described above.

#### Co-culture of PRRSV-infected moDC and sorted CD4^+^ T cells

Round-bottomed 96-well plates (Greiner Bio-One) were coated with 1.5 μg/mL of anti-CD3 mAbs (clone PPT7, IgG1) as stated elsewhere [30]. Free mAbs were removed by washing the plates three times with PBS. MoDCs cultivated for 7 days were harvested and transferred either into CD3-coated plates or non-coated plates. MoDC microcultures were either mock-infected or infected with PRRSV-2 or PRRSV-1 at an MOI of 0.02 for 2 h (as described above). In parallel, autologous PBLs were defrosted and labelled with mouse anti-porcine CD4 antibody (74-12-4, IgG2b; [27]) for 15 min at 4 °C in the fridge. Cells were washed with PBS (supplemented with 0.5% of bovine serum albumin). Cell pellets were resuspended in buffer and anti-mouse IgG2a + b MicroBeads were added (Miltenyi Biotec, Bergisch Gladbach, Germany). After incubation for 15 min at 4 °C in the fridge, cells were washed and up to 10^8^ cells resuspended in 500 μL of cold buffer. The cell suspension was applied onto LS columns (Miltenyi Biotec) for positive selection of CD4^+^ T cells by use of a Quadro-MACS system (Miltenyi Biotec). Purity of sorted CD4^+^ T cells was between 96.7 and 98.2% (mean of 97.7%). Subsequently, sorted CD4^+^ T cells were labelled with a proliferation dye (CellTrace™ Violet Cell Proliferation Kit, Life Technologies) as described elsewhere [31]. MoDCs and violet labelled sorted CD4^+^ T cells were seeded at a ratio of 1:10 in CD3-coated and non-coated plates and co-cultured for four days at 37 °C and 5% CO_2_. For each experiment, randomly selected microcultures were harvested already after 24 hpi to analyze the frequency of PRRSV-infected moDCs present in co-cultures.

Following co-cultivation, cells were harvested and stained with Live/Dead® Fixable Near-IR dye (Life Technologies) according to manufacturer’s instructions. Thereafter, cells were fixed and permeabilized using the Foxp3/Transcription Factor Staining Buffer Set (eBioscience) and subsequently labelled with rat anti-mouse/rat Foxp3-PE mAbs (clone FJK-16 s, IgG2a; eBioscience). This mAb has a well-documented cross-reactivity with porcine Foxp3 [16,32].

### Statistical analysis

Data on SLA-DR, CD80/86 and Foxp3 expression as well as CD4^+^ T-cell proliferation was analyzed for statistical significance by GraphPad Prism Version 5.01 Software (GraphPad Software, San Diego, CA, USA). Values were evaluated for normality distribution by the Kolmogorov-Smirnov test. Subsequently, data sets were analyzed using the Mann–Whitney *U* non-parametric test. *P* values below 0.05 are indicated by an asterisk (*).

## Results

### Phenotyping of moDCs

**Figure 1:**
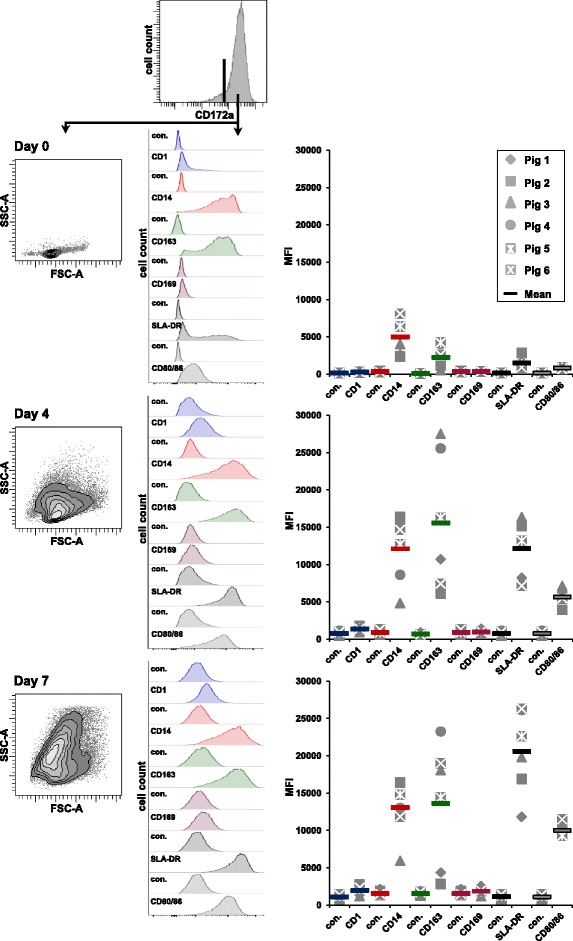
**Phenotype of porcine moDCs.** Four-color FCM including a Live/Dead discrimination dye was used to study CD1, CD14, CD163, CD169, SLA-DR and CD80/86 expression on living CD172a^high-^defined moDCs at days 0, 4 and 7 following in vitro cultivation. For each time point, live CD172a^high^ cells were gated and light scatter properties analyzed. Offset histograms show fluorescence intensities of CD1, CD14, CD163 CD169, SLA-DR and CD80/86 and their corresponding isotype controls (con.) within CD172a^high^ cells for each time point. Representative data of pig number 5 is shown. Scatter diagrams on the right show MFI (y-axis) for each analyzed surface marker and the corresponding isotype control for experiments with moDCs derived from six different pigs (indicated by different symbols). Coloured bars indicate mean values (right panel).

As outlined above, moDCs have been frequently used to study the influence of PRRSV infection on APCs and their role in co-culture experiments with PBLs or PBL-subsets. Surprisingly, not much effort has been put in the phenotypic characterisation of moDCs used in such in vitro infection experiments. Therefore, we analyzed the expression of molecules previously studied in the context of moDC generation (CD1, CD14, SLA-DR, CD80/86; reviewed in [33]) but also CD163 and sialoadhesin (CD169), which are supposed to be involved in PRRSV entry into host cells (reviewed in [34]). Accordingly, during the moDC-generation process, we analyzed gated CD172a^high^ cells for expression of these markers at day 0, i.e. immediately after plastic adherence, day 4 and day 7 by FCM (Figure 1). Voltages for photomultiplier tubes (PMT) used to analyze scattered light and fluorescence signals obtained in FCM were set to a level that allowed the investigation of the relatively dense CD172a^high^ monocytes present at day 0 but also much larger moDCs at day 7. These PMT voltage settings were kept constant throughout the time course. With the exception of CD163 and SLA-DR, the obtained fluorescence profiles did not indicate a clear distinction into positive and negative subsets (Figure 1, middle column). Therefore, we analyzed the median fluorescence intensity (MFI) obtained for the respective markers and compared it with the MFI of the corresponding isotype control samples (Figure 1, right column, scatter diagrams). CD172a^high^ cells analyzed on day 0 showed a very low expression of CD1 with a minor population of cells expressing higher levels. CD14 was highly expressed on the vast majority of CD172a^high^ cells for all animals analyzed. With regard to CD163, both CD163^−^ and CD163^+^ subsets were identified, whereas no obvious CD169 expression was found. Similarly, SLA-DR^+^ and SLA-DR^−^ CD172a^high^ cells were present; with a high variation in expression levels on individual cells. For CD80/86 moderate expression levels were observed. After 4 days of cultivation, CD172a^+^ cells increased in size and granularity and this also caused an increase in autofluorescence, as illustrated in the observed increase in the MFI of isotype controls. Compared to the fluorescence in isotype control samples, the majority of cells still showed a high expression of CD14 and CD163. The expression of CD1 was slightly increased on all cells; but they were still negative for CD169. Of note, expression levels of CD14 and CD163 varied considerably between moDCs derived from different animals. The expression levels for SLA-DR and CD80/86 had further increased compared to day 0. After 7 days of culture, moDCs still showed an increase in size and granularity compared to day 4. Expression levels of CD1 showed a further minor increase, CD14 and CD163 remained on high expression levels with a considerable animal to animal difference. There was also a minor increase for CD169 compared to the corresponding isotype control. The expression levels for SLA-DR and CD80/86 had further increased compared to day 4.

### Analysis of moDC infection rate

**Figure 2:**
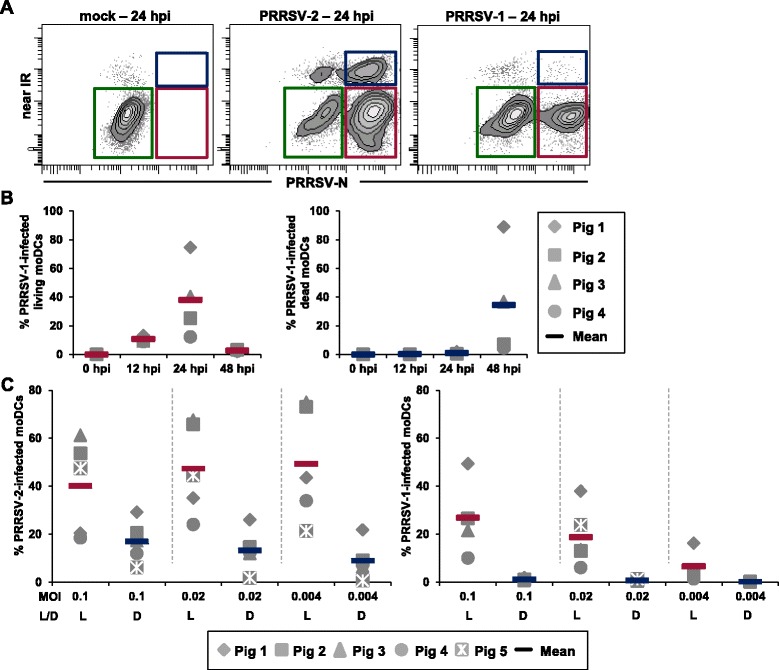
**Frequency of PRRSV-infected moDCs.** MoDCs were either mock-infected or infected with a PRRSV-2 or a PRRSV-1 strain and either cultivated for the indicated periods of time or infected with different MOI rates. **(A)** Contour plots show fluorescence intensity of PRRSV-nucleoprotein (PRRSV-N) staining versus Live/Dead discrimination dye of mock-infected (mock), PRRSV-2 and PRRSV-1 infected CD172a^+^ gated (not shown) moDCs after 24 hpi. Coloured gates indicate the following cell subpopulations: non-infected and live moDCs (green gate), infected and live moDCs (red gate) and infected and dead moDCs (blue gate). An MOI of 0.1 was used for both PRRSV-genotypes. **(B)** Scatter diagrams show the frequency of either PRRSV-1 infected live or PRRSV-1 infected dead moDCs (y-axis) after 0, 12, 24 or 48 hpi (x-axis). An MOI of 0.1 was used for infection. **(C)** Frequencies of either live (L) or dead (D) infected moDCs for different MOI rates (x-axis; separated by dashed lines) in PRRSV-2 (left scatter diagram) and PRRSV-1 infected moDCs (right scatter diagram) after 24 hpi are shown. **(B + C)** Each symbol represents data of moDCs derived from one individual animal (B: *n* = 4; C: *n* = 5). Coloured bars indicate mean values.

After 7 days of culture, moDCs were harvested, transferred into 96-well plates, and infected with a PRRSV-2 or a PRRSV-1 strain. Mock-infected cells served as control. Expression of PRRSV-N-protein and the presence of live and dead cells were analyzed by FCM. After 24 hpi, three populations of CD172a^+^ moDCs derived from PRRSV-infected microcultures could be identified: non-infected and live moDCs (green gate), infected and live moDCs (red gate) and infected and dead moDCs (blue gate) (Figure 2A). MoDCs from mock-infected cultures stayed alive and were negative for PRRSV-N-protein. In pilot experiments, we used these read-outs to analyze the frequency of infected live or dead moDCs over time, i.e. after 0, 12, 24 and 48 hpi in PRRSV-1 infected microcultures (Figure 2B). At an MOI of 0.1, the frequency of infected but live moDCs increased with time, peaking after 24 hpi (mean of 38% of total moDCs) and strongly decreased at 48 hpi. In contrast, dead infected moDCs remained at low levels for the early time points but after 48 hpi a considerable increase was found (mean of 34% of total moDCs). Having identified 24 hpi as the optimal time point to obtain a high frequency of live PRRSV-infected moDCs, we moved on to analyze the influence of different MOI rates on the frequency of PRRSV-infected moDCs (Figure 2C). MoDCs were infected with an MOI of 0.1, 0.02 or 0.004 with either the PRRSV-1 or the PRRSV-2 strain used in our study. For the PRRSV-2 strain all MOI rates gave rise to high frequencies of live infected moDCs (mean values between 40 and 50%) and lower percentages of dead infected moDCs after 24 hpi (mean values between 9 and 17%). Frequencies of live infected moDCs derived from PRRSV-1 infected microcultures were lower and showed a clear MOI dependence: mean values of live infected moDCs were 27% for an MOI of 0.1, 20% for an MOI of 0.02 and 7% for an MOI of 0.004. Of note, the frequency of infected dead moDCs stayed close to zero in PRRSV-1 infected microcultures for all tested MOI rates. In order to enable a parallel analysis of PRRSV-1 and PRRSV-2 infected moDCs, we decided to use an MOI of 0.02 for subsequent experiments as this rate caused reasonable frequencies of live and dead moDCs in PRRSV-2 infected cultures and the frequencies of PRRSV-1 infected live moDCs reached appropriate levels.

### CD163, CD169, SLA-DR and CD80/86 expression on PRRSV-infected moDCs

**Figure 3:**
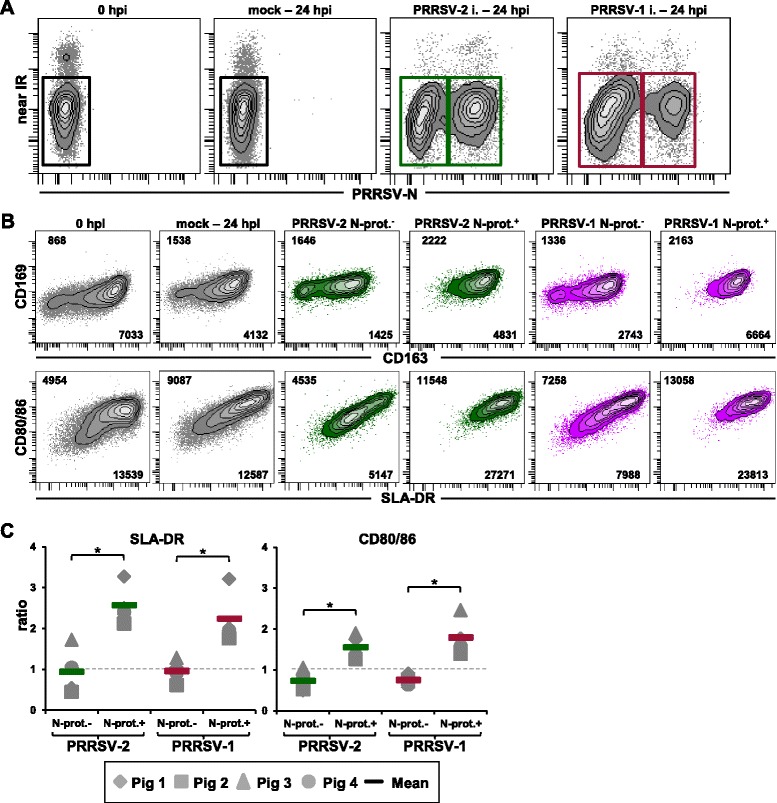
**CD163, CD169, SLA-DR and CD80/86 expression on PRRSV-infected moDCs.** Four-color FCM including a Live/Dead discrimination dye was used to study either CD163 and CD169 or SLA-DR and CD80/86 expression in moDCs before infection (0 hpi), mock-infected (mock), PRRSV-2 infected (PRRSV-2 i.) and PRRSV-1 infected (PRRSV-1 i.) CD172a^high^-defined moDCs at 24 hpi. **(A)** CD172a^high^ moDCs were gated (not shown) and analyzed for PRRSV-nucleoprotein expression (PRRSV-N, x-axis) versus Live/Dead staining (Near-IR, y-axis) at 0 hpi and 24 hpi for mock, PRRSV-2 and PRRSV-1 infected microcultures. Gates indicate PRRSV-N^−^ mock-infected live moDCs (black gates), PRRSV-N^−^ and PRRSV-N^+^ live moDCs from PRRSV-2 infected cultures (green gates) and PRRSV-N^−^ and PRRSV-N^+^ live moDCs from PRRSV-1 infected cultures (red gates). **(B)** Contour plots show expression of CD163 versus CD169 (top row) and SLA-DR versus CD80/86 (bottom row) on moDCs within the respective gates shown in **(A)**. Numbers indicate median fluorescence intensity values for CD169 and CD80/86 in the upper left corner of each contour-plot whereas numbers in the lower right corner indicate median fluorescence intensity values for CD163 and SLA-DR. Exemplary data of moDCs derived from a single animal is shown. **(C)** MFI values obtained for SLA-DR and CD80/86 in mock-infected moDC cultures were set to 1 (dashed lines) and ratios based on MFI values for SLA-DR (left graph) and CD80/86 expression (right graph) of N-prot.^−^ and N-prot.^+^ moDCs for each genotype were calculated and displayed in the respective scatter diagrams. Each symbol represents data of moDCs from one individual animal (n = 4). Coloured bars indicate mean values. Significant differences between N-protein^+^ moDCs compared to N-protein^−^ moDCs from PRRSV-2 and PRRSV-1 infected microcultures are indicated (* = *p* < 0.05).

As pointed out above, analyzes of MHC-II expression and CD80/86 have led to conflicting results for PRRSV-infected moDCs. Having identified considerable differences in the frequencies of infected/non-infected and live/dead moDCs present in PRRSV-infected microcultures depending on time point and MOI, we decided to analyze SLA-DR and CD80/86 but also CD163 and CD169 expression in infected versus non-infected moDCs present in the same culture at 24 hpi. In addition, we investigated expression of these four markers on moDCs cultures prior to infection and in mock-infected cultures 24 hpi. CD172a^+^ moDCs were gated into infected and non-infected subsets according to N-protein expression for PRRSV-2 and PRRSV-1 infected microcultures, while dead cells were excluded from the analysis (Figure 3A). N-protein^+^ and N-protein^−^ cells were then analyzed for the combined expression of either CD163/CD169 or SLA-DR/CD80/86. Accordingly, in Figure 3B the expression of these markers for six different moDC populations are shown: (i) moDCs at 0 hpi, (ii) mock-infected moDCs after 24 hpi, (iii) N-protein^−^ moDCs and (iv) N-protein^+^ moDCs present in PRRSV-2 infected cultures as well as (v) N-protein^−^ moDCs and (vi) N-protein^+^ moDCs present in PRRSV-1 infected cultures. Overall, across all analyzed populations and in accordance with data from Figure 1, the vast majority of moDCs expressed high levels of CD163, SLA-DR and CD80/86 but only very low amounts of CD169. N-protein^+^ cells present in PRRSV-1 and PRRSV-2 infected cultures had a higher expression of all four markers compared to N-protein^−^ moDCs and moDCs in mock-infected cultures, and this applied even for the very low expressed CD169 molecule. This could be verified with moDCs derived from four different animals where MFI values of SLA-DR and CD80/86 from moDCs of mock-infected cultures at 24 hpi were set to 1, and fold-increase or fold-decrease were calculated for MFI values of N-protein^−^ and N-protein^+^ moDCs present in PRRSV-infected cultures (Figure 3C). For SLA-DR N-protein^+^ moDCs showed 2.6 and 2.2 fold higher MFI values following PRRSV-2 and PRRSV-1 infection, respectively, compared to N-protein^−^ moDCs from infected cultures (*p* = 0.029 for both) and moDCs from mock-infected cultures. Similarly, 1.6 and 1.8 fold higher MFI values of CD80/86 were observed in N-protein^+^ moDCs from PRRSV-2 and PRRSV-1 infected cultures, respectively, compared to moDCs from mock-infected cultures (*p* = 0.029 for both). Of note, this molecule appeared to be slightly downregulated in N-protein^−^ moDCs from infected cultures, regardless of the virus strain used for infection.

### Co-culture of PRRSV infected moDCs and autologous sorted CD4^+^ T cells

**Figure 4:**
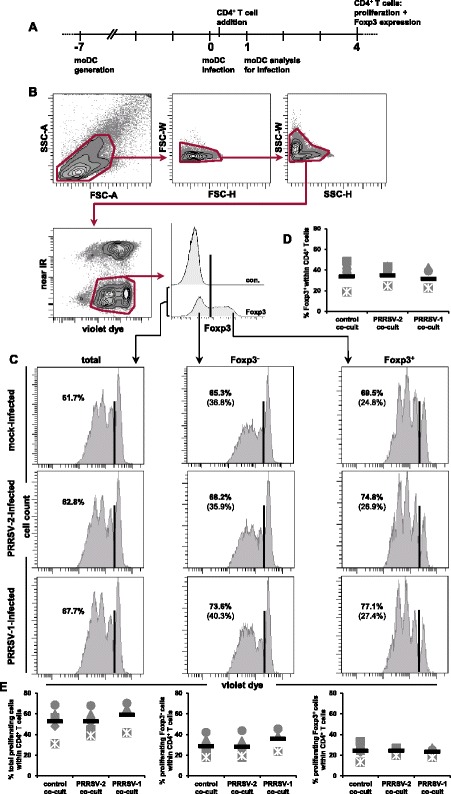
**Proliferation and Foxp3 expression in sorted CD4** ^**+**^ **T cells co-cultivated with PRRSV-infected moDCs.** **(A)** Temporal progression of experimental procedure: moDCs were harvested 7 days after the start of cultivation and infected with PRRSV-2, PRRSV-1 or mock-infected. Approximately 7 h after infection, violet-stained MACS-sorted CD4^+^ T cells were added and co-cultivated with moDCs for 4 days. One day after infection, the frequency of N-protein^+^ moDCs present in the co-cultures was analyzed. After 4 days of co-cultivation, CD4^+^ T cells were analyzed for proliferation and Foxp3 expression. **(B)** Gating strategy: doublets and dead cells were excluded by consecutive gating. Violet^high^ and violet^dim^ cells (representing non-proliferating and proliferating CD4^+^ T cells, respectively) were gated and analyzed for Foxp3 expression. Foxp3-defined subsets were gated and analyzed for proliferation. **(C)** Histograms show violet dye fluorescence intensity for total CD4^+^ T cells (left panel), Foxp3^−^CD4^+^ T cells (middle panel) and Foxp3^+^CD4^+^ T cells (right panel) in mock-infected (top), PRRSV-2 infected (middle) and PRRSV-1 infected co-cultures (bottom). Bars in histograms represent the border of proliferating and non-proliferating cells. Bold numbers in histograms indicate the percentage of proliferating cells within the respective subset; numbers in brackets show the percentage of proliferating cells within total CD4^+^ T cells. Exemplary data of CD4^+^ T cells derived from a single animal is shown. **(D)** Frequency of Foxp3^+^ cells within total CD4^+^ T cells (y-axis) for mock-infected (control co-cult), PRRSV-2 infected (PRRSV-2 co-cult) and PRRSV-1 infected co-cultures (PRRSV-1 co-cult). **(E)** Frequency of total proliferating cells (left), proliferating Foxp3^−^ (middle) and proliferating Foxp3^+^ cells (right) within CD4^+^ T cells (y-axis) for mock-infected co-cultures, PRRSV-2 infected co-cultures and PRRSV-1 infected co-cultures (x-axis). **(D + E)** Each symbol represents data of CD4^+^ T cells derived from one individual animal (PRRSV-2: *n* = 5; PRRSV-1: *n* = 3). Black bars show the respective mean values.

Having successfully established a well-defined system for the infection of moDCs with PRRSV-1 and PRRSV-2, we tested the suitability of this culture system for the previously postulated induction of Tregs derived from PRRSV-naïve animals by PRRSV in vitro [18,19]. MoDCs cultivated for 7 days were transferred either into non-coated or CD3-coated microtiter plates. Within each plate moDCs were either mock-infected or infected with PRRSV-2 or PRRSV-1. After two hours the inoculum was removed, and cultivation of moDCs was continued. Approximately 7 h after infection, autologous violet-stained MACS-sorted CD4^+^ T cells were added and cultured for 4 days (Figure 4A). Additionally, after 24 hpi, the rate of infected moDCs was analyzed to monitor viral replication under co-culture conditions. Results showed a mean of 20% of infected moDCs for the PRRSV-2 strain, and 11% for the PRRSV-1 strain (data not shown). On day 4, cells were harvested, stained for Foxp3 expression and analyzed by FCM. The gating strategy used to identify i) the frequency of Foxp3^+^ T cells, ii) total proliferating CD4^+^ T cells, iii) proliferating Foxp3^−^CD4^+^ T cells and iv) proliferating Foxp3^+^CD4^+^ T cells is illustrated in Figure 4B. This was achieved by the exclusion of doublets (FSC-W versus FSC-H and SSC-W versus SSC-H), followed by exclusion of dead cells (near IR^+^) and violet^−^ cells. Near-IR^−^violet^−^ cells were considered as surviving moDCs, since these cells had not been labelled with violet proliferation dye. Violet^dim^ and violet^high^ cells (which we considered as live CD4^+^ T cells) were then further subgated according to Foxp3 expression and the different subpopulations mentioned above were analyzed. Representative data from one experiment is shown in Figure 4C. Proliferating Foxp3^+^ cells showed slightly higher proliferation rates than proliferating Foxp3^−^ cells (Figure 4C, right panels versus middle panels, frequency numbers in bold), consistent with the concept that due to the high expression of CD25, Tregs can efficiently use IL-2 for their stimulation. However, no major differences in proliferation rates could be observed for any T-cell subset being cultivated in the presence of mock-infected moDCs, PRRSV-2 or PRRSV-1 infected moDCs. These results were consistent for T-cell/moDC co-cultures derived from several individual pigs where no statistically significant differences were found (Figure 4E). Similarly, the total frequency of Foxp3^+^CD4^+^ T cells did not change between PRRSV-infected and mock-infected co-cultures (Figure 4D). For co-cultures kept in microtiter plates without CD3-coating also no differences between PRRSV-infected and mock-infected co-cultures in regard to the analyzed cell populations could be found (data not shown).

## Discussion

Porcine moDCs have been used in many studies as an in vitro model to study PRRSV host-pathogen interactions [11-14,18] but results in regard to MHC-II expression, CD80/86 expression and induction of Tregs were often conflicting. One possible explanation to this is the use of different viral strains which often differ considerably in regard to genomic sequence and virulence, even within a particular genotype. However, we hypothesized that these differences might also be related to a suboptimal characterisation of the experimental systems used.

To overcome this, in a first step we performed a series of experiments addressing the phenotype of porcine moDCs with a marker panel consisting of CD172a, CD1, CD14, CD163, CD169, SLA-DR and CD80/86. In accordance with previous descriptions of porcine moDCs [24,33,35] the moDCs in our culture system were CD172^high^CD1^dim^CD14^+^SLA-DR^+^CD80/86^+^. We additionally analyzed expression of CD163 and CD169 since the current knowledge suggests that these two molecules are of high relevance for PRRSV-entry into the host cell (reviewed in [34]). CD163 has been described as a marker for the discrimination of different porcine monocyte subsets (reviewed in [36]). Domínguez and co-workers also investigated CD163^−^ and CD163^+^ monocytes for potential differences in regard to their differentiation into moDCs [37]. Both subsets were capable to mature into DCs but moDCs derived from CD163^+^ monocytes expressed still higher levels of SLA-DR and CD80/86 and were more efficient to induce proliferation in allogeneic T cells than moDCs derived from CD163^−^ monocytes. Of note, these authors reported that both moDC populations did not express CD163 after 7 days of in vitro cultivation. This is in contrast to our results where - albeit at different levels between moDCs from individual animals – CD163 was expressed on the vast majority of analyzed moDCs. The reasons for these discrepancies are speculative, but differences in the cultivation protocol, like the addition of TNF-α on day 5 of cultivation in the work performed by Chamorro et al. [37] may be of relevance to this.

In regard to CD169 expression monocytes analyzed in this study at day 0 did not express this molecule, which is in agreement with previous results where CD169 could be only detected on monocytes after three days of in vitro stimulation with IFN-α [38]. A moderate expression of CD169 has been reported on moDCs after 7 days of cultivation in the presence of IL-4, GM-CSF and IFN-α [39] which is different to the very low expression levels observed after 7 days in this study. The increased expression described by Revilla et al. [39] might be a consequence of the additional IFN-α stimulation as indicated by the results obtained with IFN-α stimulated monocytes [38].

Despite this overall very low CD169 expression, PRRSV replication, identified by N-protein^+^ cells, was clearly restricted to a CD163^+^CD169^dim^ phenotype as shown in Figure 3B. Thereby our results confirm the postulated role of these two molecules for PRRSV binding, internalization and RNA release [34,40-43] and consequently virus replication.

The discrimination between N-protein^+^ and N-protein^−^ moDCs also revealed that PRRSV-infected moDCs showed increased expression levels of SLA-DR and CD80/86 compared to non-infected moDCs and this applied to both PRRSV strains tested. Interestingly N-protein^−^ moDCs showed a slight downregulation of CD80/86 expression, when compared to moDCs derived from mock-infected cultures. These changes can be only observed by the distinction of infected versus non-infected cells in the same culture but not by the analysis of the expression of surface molecules for the entire cell population derived from a PRRSV-infected culture. Indeed, this finding may explain why previously published results, as outlined in the introduction, differ so widely in regard to the effect of PRRSV infection on SLA-DR and CD80/86 expression in moDCs. Remarkably, we observed a high expression of both molecules for viruses representing the two major genotypes PRRSV-1 and PRRSV-2. Moreover, our results provide no immediate hints that the expression of SLA-DR and/or CD80/86 is negatively affected by a PRRSV infection, thereby we could not corroborate a potential immuno-evasion of PRRSV by this mechanism.

The induction of Tregs in PRRSV-naïve animals was also postulated as a immuno-evasive mechanism of PRRSV. Silva-Campa et al. [18] reported an expansion of CD25^+^Foxp3^+^ lymphocytes following co-culture of PRRSV-2 infected moDCs and autologous PBLs. By using a similar experimental setup and the same PRRSV-2 strain (NVSL 97–7895) we were not able to identify an increase in Foxp3^+^CD4^+^ T cells regardless whether these cells had been stimulated with anti-CD3 mAbs (Figure 4D) or were kept in medium (data not shown). Differences in the stimulation protocol like different MOI rates (0.1, Silva-Campa versus 0.02, this study) and the time span between infection of moDCs and addition of autologous T cells (24 h, Silva-Campa versus 7 h, this study) may account for our negative results. However, as shown in Figure 2C, we did not observe major differences in the frequency of N-protein^+^ moDCs following infection with an MOI of 0.1 versus 0.02. Similarly, adding CD4^+^ T cells to moDCs 24 hpi did not change our results (data not shown). It is probably also of relevance that Silva-Campa et al. [18] used a mouse anti-human Foxp3 specific mAb clone 221D/D3 that in our hands showed no reactivity with porcine Foxp3 [16]. An induction of CD4^+^CD25^+^Foxp3^+^ T cells by moDCs infected with a PRRSV-2 isolate from Thailand was also described by Wongyanin et al. [19]. Again, these results were achieved by a protocol very similar to ours, but could not be confirmed in our experiments. It should be noted that Wongyaning and co-workers worked with a mouse anti-human Foxp3 mAb (clone 236A/E7) which has no documented history of cross-reactivity with porcine Foxp3. Moreover, the authors used a commercial fixation and permeabilization reagent kit that in our hands showed only a poor performance for the identification of antigens located in the nucleus [44].

Nevertheless, published work from Silva-Campa and co-workers as well as Wongyaning and co-workers also provided hints for increased RNA expression levels of Foxp3 in PRRSV-infected moDC co-cultures [18] and PBMCs incubated with cell lysate derived from PRRSV-infected MARC cells [19]. In a consecutive study it was also shown that moDCs transfected with N-protein coding plasmids produced IL-10 and also caused increased frequencies of CD4^+^CD25^+^Foxp3^+^ T cells in co-cultures [45]. Therefore, our results do not exclude an influence of PRRSV on Tregs. However, we think that our experiments demonstrate the difficulties in reproducing published results based on in vitro culture systems which are poorly characterised.

Moreover, as illustrated by our findings on differences in expression of SLA-DR and CD80/86 between PRRSV-infected and non-infected moDCs present in the same microculture, our work indicates that every possible effort on a detailed characterisation of the experimental systems used for PRRSV host-pathogen interaction is probably rewarded by more consistent results across different studies.
